# Safety and Reproductive Outcomes of Minimally Invasive Nerve-Sparing Surgery for Deep Endometriosis in Infertile Women: A One-Year Follow-Up Study

**DOI:** 10.3390/jpm16030166

**Published:** 2026-03-17

**Authors:** Bruna Rafaela Oliveira, Claudio Peixoto Crispi Jr., Fabio Bastos Russomano, Fernando Maia Peixoto Filho, Nilton de Nadai Filho, Marlon de Freitas Fonseca

**Affiliations:** 1 Department of Women’s Health, Fernandes Figueira National Institute for Women, Children and Youth Health, Oswaldo Cruz Foundation, Rio de Janeiro 22250-020, RJ, Brazil; brbrito14@gmail.com (B.R.O.); claudinjr@gmail.com (C.P.C.J.); fabio.russomano@fiocruz.br (F.B.R.); nilton@niltonnadai.com.br (N.d.N.F.); 2 Department of Gynecology and Obstetrics, Rio de Janeiro State University, Rio de Janeiro 20550-013, RJ, Brazil; fernando.maia.peixoto.filho@uerj.br

**Keywords:** endometriosis, infertility, minimally invasive surgery, pregnancy rate, safety

## Abstract

**Background/Objectives**: Deep endometriosis is a chronic inflammatory disease that significantly affects fertility. The objective was to evaluate the magnitude of the effect of minimally invasive nerve-sparing complete excision of endometriosis on natural conception rate in women with documented infertility. **Methods**: This pre-planned interdisciplinary retrospective observational study included 45 patients who wished to conceive naturally (spontaneous pregnancy) and were followed for 12 months after surgery. **Results**: The spontaneous conception rate was 33.3% (95% CI: 20.0–46.7) and the mean time to conception was 6.7 months. Age, body mass index, and history of infertility showed no significant differences between the success and failure spontaneous pregnancy groups, but annual income was positively associated with reproductive success (*p* = 0.022). None of the procedures needed to be converted to open surgery, required colostomy/ileostomy, blood transfusion or abdominal drains. No cases of urinary retention were observed across different levels of nerve preservation. In addition, the absence of serious surgical complications (Clavien–Dindo III/IV) supports the safety of this intervention for infertile patients. **Conclusions**: The absence of serious surgical complications in this cohort supports the concept that minimally invasive nerve-sparing complete excision of endometriosis is a safe intervention when performed by an experienced team. The results underscore the importance of exploring socioeconomic-related factors through an individualized assessment of patients who wish to conceive naturally after minimally invasive nerve-sparing surgery. Future studies focusing on personalized management of endometriosis should attempt to identify socioeconomic-related covariates that influence natural conception.

## 1. Introduction

Endometriosis is a chronic, complex inflammatory disease of multifactorial etiology that affects approximately 10% of women of reproductive age worldwide, around 190 million [1], significantly impacting their quality of life and fertility. It is estimated that between 20% and 50% of infertile women have endometriosis, with this prevalence varying according to age [2]. Characterized by the presence of endometrial tissue outside the uterine cavity, endometriosis severely impairs quality of life and often leads to one or more pelvic dysfunctions, including sexual dysfunction, infertility and bowel dysfunction [2,3,4]. Deep Endometriosis (DE), the most severe form, refers to the disease that infiltrates one or multiple organs to depths greater than 5 mm [5,6]. The main sites affected by DE are the rectovaginal septum, uterosacral ligaments, fallopian tubes, ovaries, intestine, and bladder [7,8].

Surgery remains the treatment of choice to improve health-related quality of life in cases in which medical management has been ineffective for pain relief [9,10,11] or in selected cases of endometriosis-related infertility [7]. Although the surgical approach aims to remove endometriotic implants, endometriomas, and restore pelvic anatomy to the greatest extent possible, thus contributing to the recovery of fertility [12], the direct impact of this intervention on fertility remains debated. Previous studies provide a reasonable body of evidence indicating the benefits of surgery for fertility, including improvements in postoperative pregnancy rates [13,14,15,16,17]. Despite current guidelines from the American Society for Reproductive Medicine (ASRM, 2012) [18] and the European Society of Human Reproduction and Embryology (ESHRE, 2022) [19] not recommending surgery as the standard treatment for endometriosis-related infertility, surgical intervention may be considered an alternative for patients who wish to conceive and for whom clinical treatment has been ineffective in relieving severe pain [10] or in selected cases of infertility attributed to endometriosis [7].

Focusing on the magnitude of the effect of minimally invasive nerve-sparing complete excision of endometriosis (a therapeutic approach that is constantly evolving), the objective of this study was to evaluate the rate of spontaneous conception within one year after surgery in women with previously documented infertility who desired spontaneous pregnancy.

## 2. Materials and Methods

### 2.1. Study Design

This is a pre-planned interdisciplinary retrospective observational study exploiting a long-established database of clinical information collected about each patient. The cohort consists of 62 consecutive patients referred by the patient’s personal gynecologist to the Crispi Institute for Minimally Invasive Surgery (CRISPI)—a private institution located in Rio de Janeiro, RJ, Brazil—from February 2018 through August 2021 for consideration of minimally invasive surgical treatment of DE for infertility and/or pain persisting after medical management.

In order to improve the quality of the project, the Strengthening the Reporting of Observational Studies in Epidemiology (STROBE) statement [20] and the updated Preferred Reporting of Case Series in Surgery (PROCESS) guidelines [21] were followed to the extent possible.

### 2.2. Ethics Statement

This study used data from medical records of patients who had already been treated at a center specializing in endometriosis (retrospective data collection). The research protocol was approved by an institutional review board in accordance with the 1964 Declaration of Helsinki and subsequent reviews. Patients who might have declined to take part in the study would have received the same care as the patients who gave their consent to take part in the study.

The proposal to collect and use for research purposes retrospective data from participants who had been evaluated from 1 February 2018 was first approved on 21 February 2019 by the Research Ethics Committee of the Oswaldo Cruz Institute Foundation (CEP-IFF-Fiocruz; Register CAAE number: 07885019.8.0000.5269). Next, four amendments (updates) detailing modifications in this project have also been approved, and all CEP-IFF-Fiocruz requirements have been continuously followed. The date of approval of the 4th amendment (current version) was 21 April 2023. Data collection to complete this study ended on 1 June 2023.

As this is a retrospective study, the need for specific written consent for participants who attended before 21 February 2019 was waived by the Research Ethics Committee (CEP-IFF-Fiocruz). However, written informed consent was also obtained from them. In summary, written informed consent was obtained from all participants included in this study.

This research project is up-to-date with the filing of interim reports, and the most recent approval renewal is valid through 31 December 2025.

### 2.3. Patients (Inclusion Criteria)

The 62 cases included in this study constitute a subgroup of a larger research protocol, which evaluates the follow-up of DE surgeries performed in patients admitted to the CRISPI. The cohort included women with endometriosis who, at the time of the preoperative anamnesis, had a diagnosis of infertility, defined as the failure to achieve a successful pregnancy after 12 months or more of regular unprotected intercourse [22]. The women who did not attempt to conceive (regardless of the reason), opted to maintain pharmacological ovulation suppression, became pregnant through any assisted reproductive technology (ART) or underwent hysterectomy were not included in the calculation of the spontaneous conception rate at 1-year follow-up.

Although all patients in this series were investigated in the referring services for other causes of infertility before being referred for surgery, the details of their respective investigations (before and after surgery) were not included in this study.

This study represents a specific subset of a broader research protocol dedicated to the follow-up of patients undergoing surgical treatment for deep endometriosis. Consequently, certain technical details regarding the study design, surgical protocol and the institutional database used for data collection are shared with the methodology previously described by Fonseca et al. (2025) [3] and Crispi Jr et al. (2025) [23].

### 2.4. Data Management and Systematic Collection

For over a decade, the CRISPI institute has utilized standardized protocols and electronic databases to systematically organize and store patient clinical data. These digital repositories were created to provide a uniform structure for medical record documentation. The database contains comprehensive information, ranging from patient demographics and existing comorbidities to a meticulous record of symptoms specifically linked to endometriosis.

Because most patients are referred by their primary gynecologists, the institute maintains a rigorous system to record pelvic pain levels, urinary and bowel functions, reproductive history, and quality of life indicators starting from the initial consultation and continuing through follow-ups at 6 and 12 months post-surgery. Fertility-related data are closely monitored, including the desire to conceive, the method of conception, pregnancy outcomes, and delivery details. Furthermore, the system integrates results from laboratory diagnostics and imaging reports, such as computed tomography scan, magnetic resonance imaging and ultrasound.

All clinical assessments provided by the multidisciplinary team and detailed operative reports are consistently logged in this structured format. To ensure the most current data for this study, patients who achieved a clinical pregnancy within the one-year follow-up period were contacted to complete a specialized form focused on their gestational progress.

### 2.5. Surgical Protocol

In this case series, the diagnosis of endometriosis involved four steps: medical history, physical examination, magnetic resonance imaging (MRI), and histopathological examination after surgery. The preoperative assessments were conducted on an outpatient basis, and the recommendation for surgery was made at the discretion of the Institute’s attending gynecologist (C.P.C.). The surgeries were led by the same experienced gynecologist (C.P.C.), who had more than 20 years of experience in DE surgery. The surgical team—comprising gynecological, urological, and proctological specialists, along with dedicated nursing and anesthesia staff—remained consistent throughout the series.

Summarily, the anesthetic management involved a combined regional and general protocol. This began with spinal anesthesia using isobaric bupivacaine and morphine, followed by general induction with intravenous propofol, alfentanil, and rocuronium bromide, with maintenance provided by inhaled sevoflurane. A multimodal strategy was systematically applied to control postoperative pain and nausea.

Patients were placed in the Lloyd-Davies position for laparoscopic or robot-assisted exploration of the abdomen to identify and resect lesions previously detected via MRI and physical exams. Intraoperative cystoscopy was a standard component for every patient to evaluate and manage potential endometriosis involving the ureters or bladder. When necessary, additional procedures such as hysteroscopy for intrauterine issues or non-endometriosis related surgeries (e.g., hernia repair) were performed during the same session. For intestinal involvement, the colorectal surgeon individualized the resection technique based on the size, location, and degree of stenosis of the nodules. Ovarian endometriomas were managed via cyst excision while prioritizing the preservation of ovarian tissue; ablative techniques were not utilized. Finally, all excised tissues were transferred to the pathology department for formal analysis.

### 2.6. Surgery Complications

Complications of surgery were graded according to the Clavien–Dindo classification, where grades I–II are considered minor, and grades III–V are considered major [24]. When present, important problems were highlighted and detailed for each patient. The main intra- and post-operative complications considered in this study were as follows: conversion to laparotomy, bleeding that required blood transfusion, reoperation, bowel fistula, anastomotic leakage or infections, need for a colostomy or ileostomy, and prolonged dependence on a urinary catheter due to a failure to attain normal control of spontaneous diuresis.

### 2.7. Statistics

As a descriptive study, this case series report was mathematically limited to calculating the probability of the occurrence of some events. The Kaplan–Meier method (a nonparametric method) was used to estimate the probability of spontaneous pregnancy past given time points, and the calculation of absolute risk with confidence intervals was performed using IBM SPSS Statistics Version 29.0.0.0 (IBM Corp., Armonk, NY, USA). Nonparametric tests were used to compare groups.

## 3. Results

### 3.1. Cohort Profile

**Figure 1:**
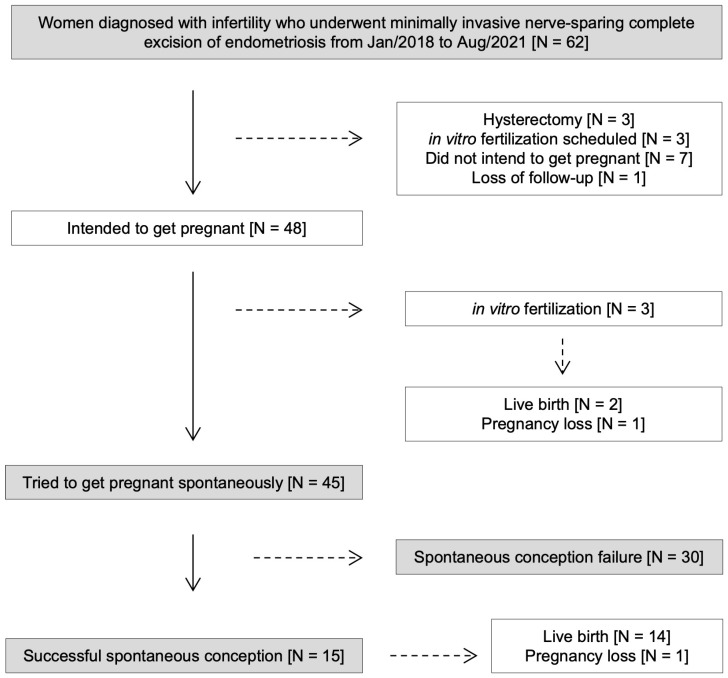
Fertility outcomes in patients surgically managed for endometriosis. Infertility: failure to achieve a successful pregnancy after 12 months or more of regular unprotected intercourse [22], or due to impaired reproductive capacity, individually or with your partner [25].

From February 2018 to August 2021, 235 consecutive DE surgeries were performed. Among them, 62 patients had documented preoperative infertility (Figure 1).

In this cohort, some cases were not included in the analysis: three women underwent hysterectomy, three were referred directly to in vitro fertilization, as previously planned by the fertility specialist, three patients were referred to in vitro fertilization after surgery due to unfavorable tubal conditions, and seven patients did not attempt to conceive spontaneously within one year after surgery. One patient whose surgery was performed in 2019 could not be located for contact; no specific reason was identified (loss of follow-up). Based on her medical records, there were no surgical complications (i.e., urinary retention, rehospitalization), and she was discharged without any problems.

Of the seven patients who did not attempt to conceive spontaneously within one year after surgery, five opted for hormonal suppression after surgery (postponing pregnancy attempts), one experienced worsening dyspareunia and reported avoiding sexual intercourse (temporarily suspending pregnancy plans), and one canceled pregnancy plans altogether.

In short, the cohort consisted primarily of Brazilian women of European descent, who were healthy, non-smokers, non-obese, and occasionally consumed alcohol up to twice a week (10%). Most had higher education levels.

### 3.2. Natural Conception After Surgery

**Figure 2:**
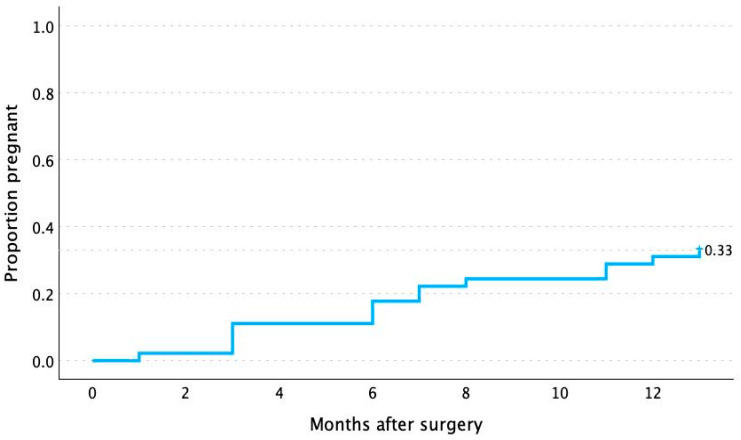
Kaplan–Meier curve showing the probability (y-axis) of becoming pregnant spontaneously over the first year after minimally invasive surgery for endometriosis: 15/45 = 33.3% (95% CI: 20.0–46.7).

Considering the 45 women with preoperative infertility who wished to conceive spontaneously during the first year after surgery, 15 succeeded, resulting in an estimated spontaneous conception rate of 33.3% (95% CI: 20.0–46.7). The average time to achieve pregnancy was 6.7 months (95% CI: 4.9–8.5); median: 6.0 months (95% CI: 3.0–9.5). Among the 15 patients who conceived spontaneously, 14 had live births (1 experienced a spontaneous miscarriage). Figure 2 shows the probability of spontaneous pregnancy over the first 12 months post-surgery in the form of a survival curve (Kaplan–Meier).

When evaluating the cohort in two groups according to the success or failure of spontaneous pregnancy within the 1-year follow-up, there were no statistically significant differences regarding age, ethnicity, marital status, education level, alcohol consumption, physical activity, or body mass index. However, annual income differed between the two groups (*p* = 0.002); the proportion of women with an annual income above $50,000 was 66.7% in the group that achieved spontaneous pregnancy and 31% in the group that did not (Table 1).

Women were grouped according to the success or failure of spontaneous pregnancy at 1-year follow-up. All women were non-smokers. Nonparametric independent-samples Mann–Whitney U test (2-sided) was used to compare groups according to ordinal variables, and Pearson Chi-square test (2-sided) was used to compare groups according to categorical variables [#Fisher’s Exact test (2-sided)]. Number of cases with missing data between brackets (question not answered). Income landmarks represent the total annual household income and were based on approximated values in December 2022. Alcohol consumption and Physical activity: days/week. BMI and age at 1-year follow-up.

### 3.3. Complications

In this series, none of the procedures needed to be converted to open surgery. None of the cases required a colostomy or an ileostomy. No case required a blood transfusion, and abdominal drains were not used. There was no case of anastomotic leakage nor any grade III or IV Clavien–Dindo complication. There were no cases of urinary retention following different levels or degrees of nerve preservation.

## 4. Discussion

### 4.1. Main Findings

This study examined the reproductive outcomes in a consecutive series of women who underwent minimally invasive surgery for DE. Among the 45 patients diagnosed with infertility who desired spontaneous pregnancy, the estimated spontaneous conception rate in the first year after surgery was 33.3%, suggesting a possible positive impact of the intervention on improving fertility. Additionally, a significant positive association, not yet fully understood, was observed between reproductive success and income (*p* = 0.002), highlighting the influence of socioeconomic factors on reproductive outcomes.

As suggested by other authors, the patient characteristics “age” and “BMI” appeared to have no significant effect on pregnancy, since the most important variables to predict a poor outcome were the duration of infertility prior to surgery and the number of previous operations [26].

### 4.2. The Potential Benefits and Risks of Surgery

Comparatively, studies by Hezer et al. [13] and Hudelist et al. [17] also demonstrated significant improvements in conception rates after similar interventions, with 62.3% of women attempting pregnancy achieving conception on average after 47.2 months, and a natural conception rate of 42.6% in 35.4 months, respectively. These results reinforce the idea that surgery for DE may be beneficial, especially for specific subgroups of patients. Additionally, the EndoRE study [27] reported a postoperative pregnancy rate of 74% in infertile patients, with 53% achieving spontaneous conception over a follow-up period of 50 to 79 months, further illustrating the potential of surgery to improve fertility.

The predictability of responses to surgical treatment is not 100%. Nevertheless, although patients should be alerted about the possibility of unsatisfactory results, endometriosis surgery provides significant improvement in deep dyspareunia [11], and this response may hypothetically result in greater success in spontaneous conception.

In the context of infertility, surgical safety is paramount. A patient seeking pregnancy should not be burdened with life-altering complications like neurogenic bladder. Highlight that your 100% success rate in avoiding urinary retention justifies the complexity of the nerve-sparing dissection. Then, concerning complications, the findings support the importance of meticulous nerve-sparing techniques in DE surgeries to minimize postoperative urinary [28,29,30] and bowel dysfunctions [28,31].

### 4.3. Income as a Determinant of Spontaneous Conception

The significant association between income and reproductive success observed in this study may suggest that women with better financial conditions have access to superior healthcare and postoperative support. Indeed, this hypothesis is consistent with studies by Farland et al. [32] and Smith et al. [33], which suggest that economic aspects can influence not only spontaneous conception rates but also access to more advanced treatments, such as in vitro fertilization. However, a statistical relationship between income and spontaneous pregnancy actually just points out (without identifying) the existence of unknown socioeconomic-related factors, which negatively affect fertility outcomes.

On 19 March 2025, the MEDLINE search strategy < pregnancy [MeSH Terms] AND endometriosis [MeSH Terms] AND infertility [MeSH Terms] AND socioeconomic factors [MeSH Terms] > identified a total of 11 publications—none of them focusing on identifying socioeconomic-related factors associated with spontaneous pregnancy rate. Hypothetically, a high-quality diet and low levels of stress due to financial problems are factors potentially associated with better outcomes (to be tested in future studies).

### 4.4. Covariates

This study also highlighted significant challenges, with 30 out of 45 patients (66.7%) not achieving pregnancy within the 1-year follow-up period. This outcome illustrates the variability in spontaneous conception rates post-surgery, which may be influenced by various factors.

Prospective cohort studies, for instance, have reported conception rates ranging from 44.4% for full-thickness disc excision to 25.6% for nerve-sparing segmental resection [30]. Furthermore, Darai et al. [14] observed that the cumulative conception rate at 52 months was nearly three times higher in patients without prior infertility, indicating how pre-surgical fertility status can significantly influence reproductive success.

Tahmasbi et al. (2025) reported a cumulative pregnancy rate of 21.4% at one year following endometriosis surgery in a retrospective study of DIE patients, including those using ART [26]. This contrasts with the findings of the present study, and this difference likely reflects distinct underlying circumstances, potentially involving a complex interplay of patient characteristics, marital dynamics, endometriosis subtypes, surgical techniques, and therapeutic support. Moreover, the potential influence of other gynecological conditions, notably adenomyosis, warrants consideration. Adenomyosis, presenting with various subtypes, distinct epidemiological profiles, and symptoms, poses significant challenges to reproductive health and is frequently overlooked or misdiagnosed as common gynecological comorbidities such as uterine fibroids and endometriosis [34].

Assessing the determinants of reproductive success after minimally invasive surgery for endometriosis is essential for understanding the complex interactions that influence the chances of conception in this patient population. These determinants encompass both female and male factors and include a range of critical considerations, such as healthy lifestyle habits, psychological support, and access to fertility treatments [35].

### 4.5. Limitations

This study’s limitations are primarily attributable to its design. The retrospective and observational nature, combined with the inherent variability of the patient cohort, restricts our capacity for causal inference regarding the effect of surgery on natural conception rates. Furthermore, the absence of consistently recorded pre-operative data on crucial parameters such as tubal status (including tubal factor), the precise anatomical distribution and severity of endometriotic lesions or adhesions, presence (and severity) of adenomyosis, and detailed surgical procedures limits the scope of subgroup analyses. In terms of external validity, the potential for selection or referral bias related to access to care warrants consideration. The underrepresentation of women from very low socioeconomic backgrounds and those identifying as non-European descent in our sample should be acknowledged as a limitation to generalizability [36].

Another significant limitation of this study is the lack of consideration for couple of factors, known to significantly impact conception rates and crucial for interpreting the results (including male conditions, hormonal status, and post-surgical ovarian reserve). Although the mechanisms through which endometriosis leads to infertility remain unclear, proposed ones mainly include not only pelvic deformity anatomy, altered endocrine function and endometrial receptivity changes, but also decreased quality and quantity of oocytes [37]. Given that male factors (abnormal semen quality or sexual dysfunction) may be implicated in approximately half of subfertile couples [38], the absence of detailed investigation data for the partners in this case series prevents a complete exclusion of some potential male subfertility. Furthermore, the relatively small sample size may restrict the generalizability of these findings to the wider population of women with endometriosis-associated infertility.

The analysis of income data suggests that socioeconomic factors may have influenced the outcomes; however, the study did not control for other social and behavioral variables that could also affect fertility.

This was a retrospective analysis of an intervention (a case series report). Nevertheless, the method was stronger than usual retrospective studies because it included a very consistent preplanned process for data capture.

Finally, despite being quite common in before-and-after studies, our team recommends being very cautious when comparing data because endometriosis is a highly individualized condition, as are results and adverse consequences of its treatment [39].

### 4.6. Challenges for a Personalized Therapeutic Planning

While distributed throughout the population, endometriosis is not a uniform condition [40]. Clinical presentations vary greatly, not only in terms of the quantity, anatomical distribution and depth of lesions, but also in terms of the type and intensity of painful symptoms (both cyclic and acyclic) [41]. Similarly, lesion type, number, and severity showed no conclusive link with conception [42], whereas endometriosis-related infertility usually presents heterogeneous responses to different pharmacological, surgical and ART treatments. This variability may occur for several reasons. Some are not yet well known; others are completely unknown.

Regarding fertility outcomes (focus of this study), the surgeon characteristics to perform different types of endometriosis surgery represent a point of concern; experience is even more required when large (both wider and deeper) resections are necessary in the most complex cases of DE [43].

Another controversial issue is the ideal approach to endometriomas [44]. The paradox of the management of endometriomas in infertile patients is that approximately 60% of women with endometriomas will need ART in the future, and, therefore, the procedure that can be offered to enhance spontaneous pregnancy rates in these patients can also worsen their future assisted reproductive outcomes [45]. Oocyte cryopreservation represents a potential fertility preservation strategy among women planning to undergo surgery (even those who have undergone previous procedures), to secure additional oocytes in advance [46]. However, it should not be offered to all women suffering from endometriosis; patients must have all the information to decide, in full knowledge of the facts (pros and cons), whether or not to perform an oocyte cryopreservation [47].

The existence of socioeconomic-related factors that impact fertility is a problem, as people diagnosed with infertility still have restricted access to ART due to its high costs, especially for low-income populations [48].

Data on obstetric outcomes of spontaneous conceptions after DE surgery are still scarce [49]. Then, although poor reporting of case series undermines critical appraisal, assessment of external validity and whether, for instance, surgeons should change their practice [21], the important ethical limitations involved in randomized studies and the difficulty in defining control groups make good observational studies fundamental to understanding the outcome of spontaneous pregnancy following surgery for DE in infertile women.

## 5. Conclusions

Our results reinforce that minimally invasive nerve-sparing complete excision of endometriosis in women with infertility is a highly safe procedure that may improve spontaneous conception rates in the first year after the procedure, as 1/3 of the patients became pregnant during the first year of follow-up. The significant positive association between income and reproductive success suggests that socioeconomic-related factors may play an important role in pregnancy outcomes, highlighting inequalities that can affect both access and treatment outcomes. Our findings support a broader individualized diagnostic and therapeutic approaches considering not only the usual clinical and laboratory parameters, but also lifestyle habits and socioeconomic conditions, factors that could negatively affect fertility of women with DE. Future studies focusing on personalized management of endometriosis should attempt to identify the main socioeconomic-related covariates that influence reproductive success after minimally invasive nerve-sparing surgery.

## Figures and Tables

**Table 1:** Demographic characteristics.

		Success (N = 15)	Failure (N = 30)	*p* Value
		N (%)	N (%)	
Ethnicity	Asiatic	0 (0)	1 (3.3)	0.789
(self-reported)	African-descent	1 (6.7)	4 (13.3)	
	European-descent	11 (73.3)	19 (63.3)	
	Mixed	3 (20.0)	6 (20.0)	
Partner	Never	1 (6.7)	1 (3.3)	0.532
(stable relationship)	Not currently	0 (0.0)	2 (6.7)	
	Yes	14 (93.3)	27 (90.0)	
Schooling	High school	0 (0.0)	1 (3.3)	0.733
(completed degree)	High school (completed)	1 (6.7)	4 (13.3)	
	College	1 (6.7)	2 (6.7)	
	College (completed)	6 (40.0)	7 (23.3)	
	Post-grad	2 (13.3)	2 (6.7)	
	Post-grad (completed)	5 (33.3)	14 (46.7)	
Income (U$/year)	<10,000	0 (0.0)	1 (3.4) [1]	0.022
	10 to 20,000	3 (20.0)	3 (10.3)	
	20 to 50,000	2 (13.3)	16 (55.2)	
	50 to 100,000	4 (26.7)	7 (24.1)	
	>100,000	6 (40.0)	2 (6.9)	
		10th/Med/90th	10th/Med/90th	
Alcohol consumption		0/0/2 [2]	0/0/2 [2]	0.589
Physical activity		0/0/3	0/1/3	0.082
Height (cm)		1.54/1.62/1.69	1.52/1.62/1.72	0.866
BMI (Kg·m^−2^)		20.0/23.0/29.6	19.9/25.0/31.3	0.546
Age (years)		30.1/33.9/39.2		0.531

## Data Availability

The original contributions presented in this study are included in the article. Further inquiries can be directed to the corresponding author.

## Abbreviations

The following abbreviation is used in this manuscript:

DE	Deep Endometriosis
